# Construction and Application of Hepatocyte Model Based on Microfluidic Chip Technique in Evaluating Emodin

**DOI:** 10.3390/nu14132768

**Published:** 2022-07-05

**Authors:** Di Chen, Jiyong Yin, Zhuo Yang, Wen Qin, Junsheng Huo, Jian Huang, Jing Sun, Wei Piao

**Affiliations:** National Institute for Nutrition and Health, Chinese Center for Disease Control and Prevention, Beijing 100050, China; chendi@ninh.chinacdc.cn (D.C.); yangzhuo@ninh.chinacdc.cn (Z.Y.); qinwen@ninh.chinacdc.cn (W.Q.); huojs@ninh.chinacdc.cn (J.H.); huangjian@ninh.chinacdc.cn (J.H.); sunjing@ninh.chinacdc.cn (J.S.); piaowei@ninh.chinacdc.cn (W.P.)

**Keywords:** microfluidic chip, raw materials of health food, emodin, hepatocyte model, evaluation

## Abstract

The current cytological evaluation technique of health food raw materials does not entirely meet the needs of evaluating health food. Our study adopted the microfluidic chip technique for the first time to construct a hepatocyte model of evaluating emodin, which was composed of a human hepatocellular carcinoma cell (HepG2) and microfluidic chip. The mixed glue of a model with rat tail collagen type I (1.3 mg/mL) + gelatin (7.5%) was used to simulate the microenvironment of a cell. The validity of this model was evaluated by cell proliferation activity and cell staining, and the toxicity of emodin was evaluated by a series of metabolic indicators on this model. The results indicated that the repeatability of the constructed hepatocyte model was favorable, with a coefficient of variation (CV) of 2.8%. After emodin continuously was exposed for 48 h, the cell inhibition was obvious at 100 and 200 μM, and the number of dead cells gradually increased with the increasing of emodin concentration, and the difference of BUN was significant between the emodin group and blank group (*p* < 0.05). The constructed model has a favorable applicability in evaluating emodin. This study provides an important platform and a potential in vitro alternative model for assessing and predicting the health effects of health food.

## 1. Introduction

Since the 1980s, health food, as a kind of food that is suitable for certain groups of people and has a special role in regulating human body functions but not for the purpose of treating diseases, has been widely concerned by people at home and abroad [1]. Since it can be taken for a longer time and can benefit more people, it might produce a greater impact on human health if there are uncertain factors in the functional components of that [2].

**Figure 1:**
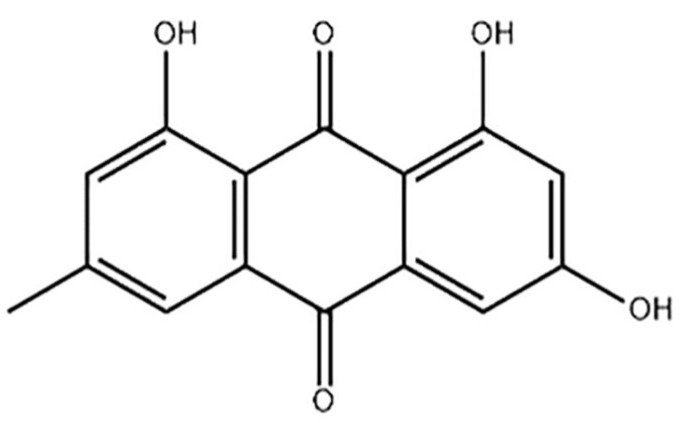
The structural formula of emodin.

Emodin (1, 3, 8-trihydroxy-6-methylanthraquinone), as one of nature’s anthraquinone compounds, mainly exists in *Rheum officinale*, *Fallopia multiflora* (*Thunb.*) *Harald*, *Reynoutria japonica Houtt.* and *Folium sennae* [3], Chemical Abstracts Service (CAS) number 518-82-1, and molecular formula C_15_H_10_O_5_ (structural formula in Figure 1) and relative molecular mass is 270.23 [4]. As one of the main ingredients of various raw materials of health food, emodin has been widely concerned and studied due to its anti-inflammatory, antibacterial, antiviral and other health care effects. Multiple studies have shown that emodin has dual effects on the liver, with a protective effect in small doses and toxicity in large doses [5]. The results of Uhp liquid chromatography-tandem mass spectrometry once was used to detect the distribution of emodin in tissue and showed that emodin was mainly distributed in liver tissues whether it was introduced into the body by intravenous injection or oral administration, which was further speculated to be one of the reasons for the hepatotoxicity caused by emodin [6,7].

In vitro experiment results showed that the damage of L02 liver cells increased and the survival rate of that decreased gradually with the increasing of emodin dose (2.5–20 μmol/L) [8]. The survival rate of HK-2 cells was decreased by 29% and 45% as inducing caspase-3 apoptosis pathway when these cells were treated with 40 μM and 80 μM emodin for 24 h [9]. In conclusion, as risk substances with bidirectional effects in health food raw materials, emodin will be toxic at a high dose, but will play an effective role in nutrition and health care at an appropriate dose. Therefore, it is very important to evaluate the safety and effectiveness of emodin doses.

In recent years, the industry of health food was gradually developed and improved with the successive enhancing of the level of modern scientific research, but the researches involved to the evaluation of raw materials of health food, the use of dose limit and other aspects were still relatively lacking, which directly resulted in the serious shortage of theory and technology of evaluation on health food [10].

At present, the evaluation methods of emodin are mainly conventional animal models and cell plate cultures, but the existing conventional methods cannot meet the requirements of evaluating emodin of health food in authenticity and validity. Conventional animal model is controversial in evaluation because it is time-consuming, uncontrollable in some aspects, unquantifiable, unable to perform multi-parameter studies and has species differences, which makes it difficult to extrapolate the results to humans [11,12].

The advantages of cell culture method are that special human cells in target tissues can be specifically selected. Cytological experiments based on petri dish and well plate once were considered as favorable candidates with simple and effective characteristics in predicting human responses to drugs by evaluating cell viability and detecting metabolites. However, conventional cell culture in vitro, which is widely used to replace animal experiments, has its limitations because it is in a macroscopic static petri dish and does not conform to the real microenvironment of existing coordination and inhibition among different cells in vivo [13,14,15,16].

In view of the disadvantages of these methods, microfluidic chip technology, as a rapid, high and new technology, can be better than them because it can complete the accurate control of cell growth, and has a series of advantages including fast analysis speed, high throughput, low energy consumption, small pollution, real-time dynamic observation, and microscopic simulation of human real characteristics, through a variety of designs and channel parameters [17,18]. In extensive cell biomedical application, such as disease prevention and diagnosis, drug screening, environmental detection, food safety, judicial identification, aerospace and other fields related to human life quality and safety, it provides a promising platform and highlights broad application prospects [19,20,21,22,23,24,25,26,27].

Although microfluidic technology has been applied in many research fields at present, it is a pity that a literature review showed that there were few applications and in-depth studies on the combination of microfluidic technology, nutritional efficacy and health product functions. In such circumstances, this research intends to construct a bionic hepatocyte model, which is based on the optimization conditions of microfluidic cell culture in the early stage of our research team, and to verify the adaptability and functionality of the hepatocyte model. The exposed analysis of emodin on the microfluidic hepatocyte model was conducted effectively at the same time.

The successful construction of in vitro hepatocyte model, which combined the microfluidic chip technology with the evaluation of raw materials of health food, not only can solve the disadvantages of conventional evaluation methods, but also can more realistically and appropriately simulate the condition of the human body.

## 2. Materials and Methods

### 2.1. Materials and Instruments

In this study, the main materials include: Emodin (Tokyo chemical industry Co., Ltd., Tokyo, Japan);Endothelial Cell Medium (ECM) (ScienCell biotechnology company, San Diego, CA, USA); Dulbecco modified Eagle’s minimal essential medium (DMEM) (Gibco Life Technologies, Grand Island, NY, USA); Trypsin-EDTA Solution (0.25%) and rat tail type I collagen (Beijing Solarbio Science & Technology, Co. Ltd., Beijing, China); Fetal bovine serum (GEMINI company, Woodland, CA, USA); Gelatine (Shanghai Aladdin Biochemical Technology Co., Ltd., Shanghai, China); Live/dead cell viability/toxicity test kit (Nanjing KeyGen Biotech. Co., Ltd., Jiangsu, China); Cell count kit (CCK-8) (Beyotime Biotechnology Co., Ltd., Shanghai, China); Human lactate dehydrogenase (LDH), aspartate aminotransferase (AST) and urea nitrogen (BUN) kits (Nanjing Jiancheng Bioengineering Research Institute Co., Ltd., Nanjing, China). The main instruments include: CellASIC ONIX2 (Merck & Co Inc, Darmstadt, Germany); EVOS M7000 Imaging system (Invitrogen Life Technology Co., Ltd., Carlsbad, CA, USA); ECLIPSE TS100 (Nikon Corporation, Tokyo, Japan); Thermo Scientific LegendMicro (Thermo Scientific Co., Ltd., Waltham, MA, USA); SpectraMax I3X Enzyme marker (Molecular Devices Instruments Ltd., San Jose, CA, USA).

### 2.2. Cell and Culture

Human hepatocellular carcinoma cells (HepG2, item no.:TcHu72) were purchased from Shanghai Cell Bank of Chinese Academy of Sciences in this study. HepG2 cells were cultured in DMEM cell culture medium (with 10% fetal bovine serum) and the temperature was set to 37 °C and the CO_2_ concentration was 5% in cell culture flask. When the growth and fusion reached 80–90% of the area, the cells were digested with 0.25% trypsin and sub-cultured. After several days of culture, the next step of microfluidic chip construction could be carried out only when the cell amount reached the requirement.

### 2.3. Preparation of Emodin Mother Liquor

Firstly, 5.40 mg emodin mother liquor was accurately weighed and dissolved in DMSO, and its final concentration was set to 40 mmol/L. Then, solution was slowly blown with pipette, and aliquot in EP tubes, clearly marked and sealed with sealing film, and stored at 4 °C for later use.

### 2.4. Basic Characterization and Pre-Treatment of Microfluidic Device

**Figure 2:**
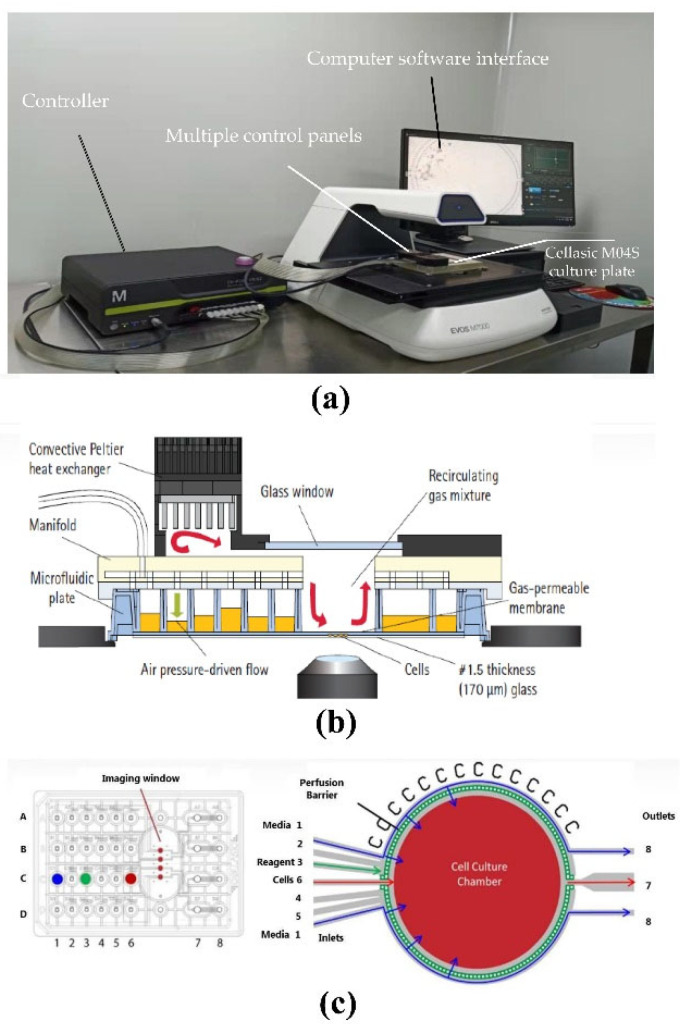
Cellasic microfluidic device dynamic cell culture structure diagram. (**a**) Microfluidic system device. The system includes a computer software interface, controller and multiple control panels. (**b**) View of system in dynamic cell culture microscope; (**c**) Working principle of Cellasic M04S culture plate.

In this experiment, the CellASIC microfluidic cell chip laboratory and Cellasic M04S cell culture plate were used together. The cell chip laboratory can integrate cell culture and functional analysis to achieve maximum dynamic cell culture, so as to achieve the purpose of restoring the natural cell growth environment in the body, and overcome the limitations of conventional cell culture to the greatest extent. The gas and temperature controllers of the control system can be directly connected to the four cell culture chambers of Cellasic M04S for individual culture and analysis. The customized Cellasic M04S cell culture plate (Figure 2c) [28,29] includes 8 columns and 4 rows (A–D); between columns 6 and 7 are the “imaging windows” of 4 cell culture chambers. The hole in column 1 of A-D is set as the cell culture medium area, the hole in column 2–5 of A–D can be set as the concentration gradient drug sample or other solution area, and the hole in column 6 of A-D is set as the cell loading hole. The specific structure of the microfluidic chip is as follows (Figure 2b) [28,29]. The pressure and flow controller of the device is composed of a vacuum pump, an electronic pressure controller and a solenoid valve. The vacuum pressure is controlled by a computer operation with customized software to control the liquid flow rate in the sample hole, and the continuous airflow enters the cell growth area through diffusion (Figure 2a). Before the microfluidic chip experiment, the internal microenvironment of the chip was pretreated, and the control board, culture plate chip, rubber pad, etc. were sterilized by ultraviolet irradiation for 1 h. Before using the chip for cell culture, it was necessary to pass PBS into the cell culture channel of the chip through a micro-syringe pump for cleaning before use [28,29].

### 2.5. Cultivation and Model Construction of Hepatocytes on a Microfluidic Chip

On the first day of the experiment, the adherent HepG2 cells that had been cultured well in ordinary culture flask were digested with 0.25% trypsin, and the reaction was terminated in DMEM medium with 10% fetal bovine serum. After collection, centrifuge as 1000 revolutions per minute (RPM) for 3 min, remove the supernatant, add 100 μL of DMEM medium with 10% fetal bovine serum to resuspend and count the cells, and dilute until the concentration of cells to 20 × 10^6^ cells/mL after constant volume. Finally, the optimal mixed glue (7.5% gelatin + 1.3 mg/mL rat tail collagen type I + 0.1% NaOH + 10 × PBS), which had been optimized by the laboratory team in previous research, was used to establish and simulate the cell matrix environment, and the HepG2 cells was evenly configured with mixed glue solution to a final concentration of 2.54 × 10^6^ cells/mL After opened the mini-fold cover plate, which connect with microfluidic cell chip laboratory and is a part of that, 50 μL 2.54 × 10^6^ cells/mL HepG2 cells were added to A6, B6, C6 and D6 of Cellasic M04S cell culture plates, respectively, and 300 μL ECM was added to A1, B1, C1 and D1 to provide culture materials for cells in cell culture chamber through the gravity action of ECM fluid. Then, the Cellasic M04S cell culture plate was sealed with mini-fold cover plate. As the cell loading conditions included 0.26 Psi pressure, 6s and 2–3 times, the HepG2 cells in each hole of 6th column were simultaneously loaded in corresponding each cell culture chamber. Under 37 °C and 5% CO_2_, and the cells were continuously cultured for 24 h. After the addition of HepG2 cells, each cell culture chamber were photographed by EVOS M7000 Imaging system.

### 2.6. Emodin Induced HepG2 Cell Toxicity Assessment

In the second day post adding HepG2 cells, cell culture medium containing emodin with different concentrations (0, 25, 50, 100, 200 μM) was added into the hole in column 2 of A-D more times. Then, they were respectively added into the cell culture chamber through pressure pump for continuous perfusion with 1.3 Psi pressure. Subsequently, a 48 h toxicity evaluation experiment was conducted on different groups. After two days of emodin stimulation, 120 μL CCK8-prepared solution (50 μL CCK-8 reagent and 500 μL DMEM medium without serum) was added into the hole in column 3 of A-D under the continuous perfusion with 3.8 Psi pressure, and cells were incubated continuously at 37 °C and 5% CO_2_ for 3 h. The liquid in the A7–D7 column was sucked out and collected in a 96-well plate post 3 h. The absorbance was measured at 450 nm with a microplate reader, and 3 repeated wells were measured in parallel for each concentration. The cell viability of the HepG2 cell with a different emodin concentration was calculated as the formula (1).

Cell viability (%) = [A (dosed) – A (blank)] × 100/[A (0 dosed) – A (blank)](1)

A (dosing): OD value of the well with cells, CCK-8 solution and drug solution

A (0 dosing): OD value of the well with cells, CCK-8 solution but no drug solution

A (blank): OD value of the hole without cells

### 2.7. Influence of Emodin on Function of Liver Model

After the established hepatocyte model received 48 h intervention with different doses of emodin, cell metabolites in A7–D7 holes were collected for biochemical analysis of emodin intervention. The analysis indicators of metabolic fluid included aspartate aminotransferase (AST), urea nitrogen (BUN) and lactate dehydrogenase (LDH), which mainly focused on the effects of different concentrations of emodin on the metabolic functions and metabolites of liver cell. The specific detection of AST, BUN and LDH were carried out according to the kit instructions.

### 2.8. Assessment of Live and Dead Liver Cells

After CCK8 reactant was sucked out, PBS was added into the hole in column 5 of A–D (130 μL/well) after the perfusion was set for 20 min under 5 Psi pressure, then the chambers were rinsed by PBS. Subsequently, the mixed live-dead (AM and PI) reagent (AM:2 μM, PI:8 μM) were added into hole in column 4 of A-D (100 μL/well) and they were added into each chamber after the perfusion was set for 40 min under 3.3 Psi pressure. Then, the cells in chamber was double-stained with AM and PI, simultaneously. After staining, the cells were observed and photographed under fluorescence microscope to evaluate the survival and death of these cells.

### 2.9. Data Analysis

All experiments were repeated three times, and all data met normality and equal variances. The measurement data were expressed as means ± standard deviation. Student’s *t*-test was used in the experiment of the growth status of hepatocytes for the comparison between two groups, and a one-way analysis of variance (ANOVA) followed by a least significant difference (LSD) test was used in the analysis of hepatocellular toxicity of emodin and the effects of emodin on metabolic indicators for multiple comparisons. A two-sided *p* value < 0.05 was considered statistically significant. Microsoft Excel 2010 (Mircosoft Inc., Redmond, Washington, DC, USA) and SPSS19.0 (IBM, Armonk, NY, USA) were used in this research for statistical analysis, which were supported by Windows 7 operating system (Mircosoft Inc., Redmond, Washington, DC, USA).

## 3. Results

### 3.1. Determination of Basic Parameters for Establishing Microfluidic Hepatocyte Model

In previous research from our team, we referred to a large number of literatures and found that most of the gel materials have good biocompatibility. Rat tail collagen type I, as an extracellular matrix for new cell culture, is often used in in vitro cell culture experiments. In particular, the three-dimensional (3D) culture of cells can be achieved, in addition to interacting with integrin receptors to regulate gene expression, it can also support cell growth and differentiation [30]. As other biocompatible hydrogels, gelatin has excellent physical properties, including affinity, high dispersibility and dispersion stability [31]. The inappropriate concentration of the mixed glue would lead to incorrect results including the blockage of cell channels in Cellasic M04 S cell culture plate, which was caused by the high concentration of mixed flue, and the inability of constructing a simulation environment of cell 3 D culture, which were caused by low concentration of mixed flue. Therefore, we innovatively selected rat tail collagen type I (1.3 mg/mL) and gelatin (7.5%) as the concentration of mixed glues of hepatocyte model. The determination of these parameters also had been important innovation points in this study.

### 3.2. Growth Status of Hepatocytes in Microfluidic Chip Platform

**Figure 3:**
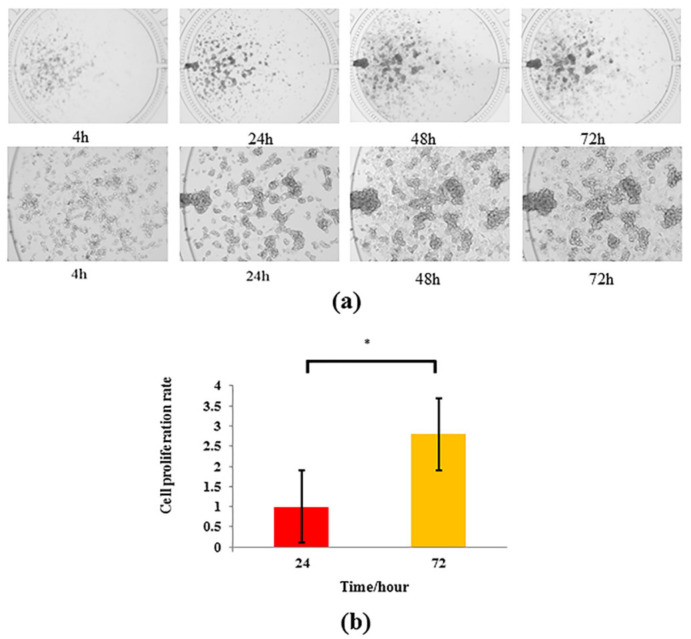
Growth status of HepG2 cells cultured on microfluidic chip. (**a**) Growth of HepG2 cells under (×4) and (×10) lenses for 4 h, 24 h, 48 h and 72 h. (**b**) Proliferation rate of HepG2 cells at 24 h and 72 h (*n* = 3). * *p* < 0.05 is compared with 24 h group.

Figure 3 indicates that HepG2 cells in chamber could complete adherent growth on the wall of the chamber at 4 h after they were loaded into the chamber, and these cells gradually presented globular and stereoscopic proliferation, and were cell clusters of distribution form or visible spindle shape because they dissolved in mixed glue with gelatin and rat tail collagen type I. After 72 h culture, the cell morphology changed with the increasing of time, and they were full and flat in the culture chamber. After 72 h cell culture, the proliferation rate of CCK8 cells was nearly three times that of 24 h (*p* < 0.05), and the coefficient of variation of CCK8 cell proliferation index was 2.8%. The results of cell proliferation rate calculation showed that microfluidic chip and liver cell module environment were suitable for the growth of liver cells, and the cell proliferation rate was significantly improved with good repeatability and low dispersion degree, and the model was stable.

### 3.3. Analysis of Hepatocellular Toxicity of Emodin Based on Microfluidic Chip

**Figure 4:**
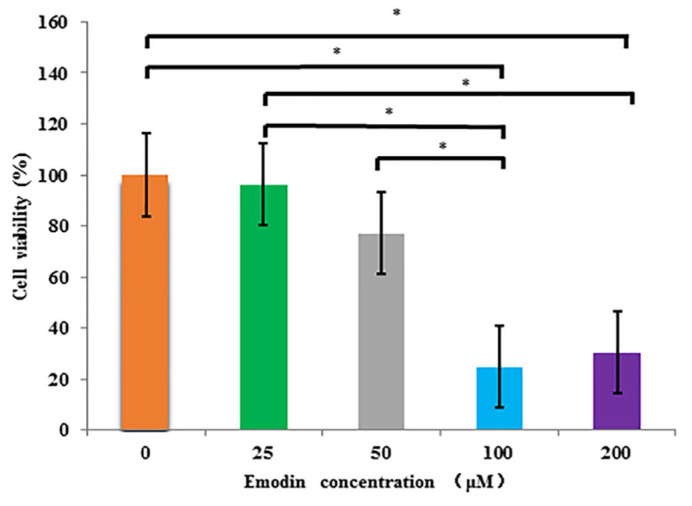
The viability of HepG2 cells exposed to Emodin at different concentrations (0–200 μM) in microfluidic culture by CCK8 kit (*n* = 3). * multiple comparisons, *p* < 0.05.

After microfluidic exposed culture with emodin at different concentrations (0, 25, 50, 100, 200 μM) for 48 h, the average results of each group are shown in Figure 4. According to the cytotoxicity results of each administration group, the survival rate of HepG2 cells gradually decreased with the increasing of emodin concentration, and the cell survival rate decreased to 24.8% and 30.4% at 100 and 200 μM, respectively, and that was the lowest at 100 μM. The ANOVA results indicated that the differences of survival rate between blank group (0 μM) and 100 μM group, and between blank group (0 μM) and 200 μM group were significant (*p* < 0.05), respectively. The differences of that between 25 μM group and 100 μM group, and between 25 μM group and 200 μM group were significant (*p* < 0.05), respectively. In addition, the difference of that between 50 μM group and 100 μM group was significant (*p* < 0.05).

### 3.4. Dead-Live Staining Results: The Effect of Emodin on Hepatocyte Viability in Microfluidic Chip

**Figure 5:**
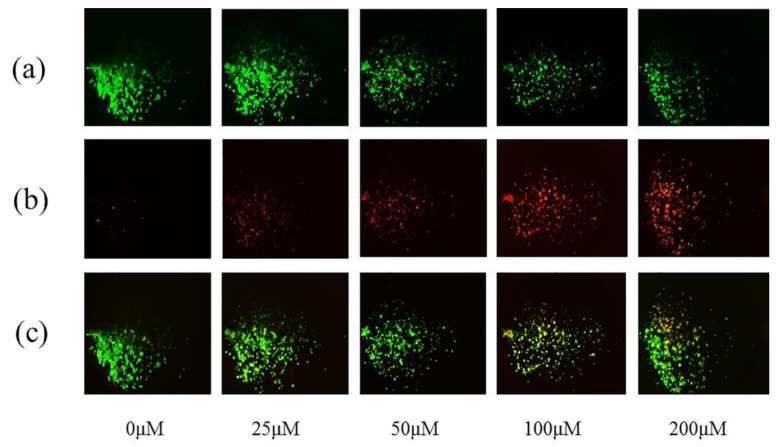
Fluorescence images of HepG2 cells exposed to emodin (0, 25, 50, 100, and 200 μM) for 48 h by live/dead assay kit. (**a**) Fluorescence images of living cells at different concentrations of emodin (0, 25, 50, 100, and 200 μM). (**b**) Fluorescence images of dead cells at different concentrations of emodin (0, 25, 50, 100, and 200 μM). (**c**) Merged fluorescence images of dead and alive cells.

After 48 h emodin exposed, the cells were tested with live and dead cell staining kit. After 30 min of incubation with dead-live dye, the results were shown in Figure 5. With the increasing of emodin concentration (0, 25, 50, 100, and200 μM), the number of dead cells (marked by red) increased gradually (Figure 5b), the number of living cells (marked by green) decreased gradually (Figure 5a), and the degree of apoptosis increased from 0 μM to 200 μM, especially at 100 and 200 μM.

### 3.5. Effects of Emodin on Metabolic Indicators of Hepatocyte Model Based on Microfluidic Chip

To verify the hepatotoxicity of emodin on hepatocytes, HepG2 cells were exposed with different concentrations (0, 25, 50, 100, 200 μM) for 48 h. Biochemical test results of the metabolic indicators showed that the AST level decreased at 25 μM while showing an increasing trend within the range of 50–100 μM; despite that, the differences among different groups was not significant (*p* > 0.05). When the concentration was 200 μM, the LDH level decreased to the lowest point, but there was no significant difference with the blank group (*p* > 0.05). In addition, the differences of LDH level among different concentrations also were not significant (*p* > 0.05).

**Figure 6:**
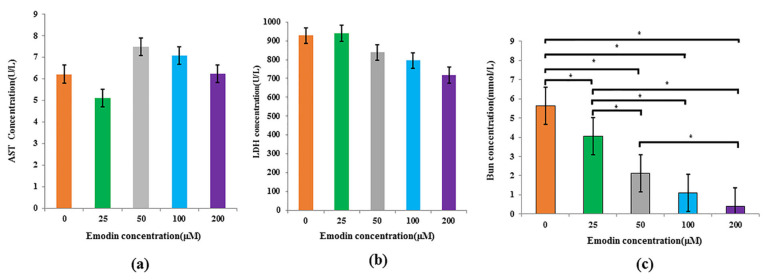
Effects of emodin at different concentrations (0, 25, 50, 100, and 200 μM) on metabolic indexes of HepG2 cells model based on microfluidic chip. (**a**) The AST activity; (**b**) LDH level; (**c**) BUN content; * multiple comparisons, *p* < 0.05.

However, compared with the blank group, the BUN level of metabolic fluid decreased significantly with the increasing of emodin concentration (*p* < 0.05). In addition, the difference between 25 μM group and 50 μM group, between 25 μM group and 100 μM group, and between 25 μM group and 200 μM group were significant (*p* < 0.05), respectively. The difference between 50 μM group and 200 μM group was also significant (*p* < 0.05) (Figure 6).

## 4. Discussion

In this experiment, microfluidic technique was selected as the research platform to design and construct a functional hepatocyte model, so as to evaluate the effect of emodin with a bidirectional role on the activity of HepG2 cells and evaluate the hepatotoxicity caused by it. The establishment of this platform not only combined microfluidic technology with nutritional efficacy and health product functions at the first, but also might be used in more nutritional function evaluations. This hepatocyte model is different from the previous direction of microfluidic research, which mainly focused on the design and manufacture of microfluidic chips.

This study adopted the proven technique that included CellASIC^®^-ONIX2 microfluidic cell chip laboratory and CellASIC M04S Mammalian cell culture plate, which are already relatively mature in the relevant field, and which can greatly optimize the environment of cell culture, and monitor the changes of cell perfusion, temperature and gas in real-time, track the response of individual cell, and achieve really dynamic cell analysis, so as to obtain the more bionic, accurate and reliable evaluation results for emodin.

In order to better simulate the metabolic function of hepatocyte in the human body, we added rat tail collagen type I and gelatin, a natural extracellular matrix with a 3D cell culture medium growth environment, into the HepG2 cells. The innovative combination of the two materials was not found in other literatures, and the research about how to combine different concentrations of gelatin also was relatively scarce. Therefore, our experimental team adopted a large number of explorations, comparisons and condition optimizations with previous research to determine that the combination of rat tail collagen type I (1.3 mg/mL) + gelatin (7.5%) can simulate the interstitial tissue among hepatocytes and provide a stable extracellular microenvironment for them. This combination not only can enable these cells to achieve better proliferation and distribution, but can also avoid the block of microfluidic channel of the culture chamber, which is caused by too high a concentration of gelatin.

In the modeling stage, we observed and determined the morphological changes of hepatocytes through cell staining, which could identify live and dead cell viability. The experimental results showed that the hepatocytes grow steadily, the proliferation rate after 72 h was nearly three times that after 24 h under the modeling parameters, and the cells were in favorable shape. In 72 h, the hepatocytes can generally complete cell division and cell spreading with the extension of time, which could also indicate that the combination of rat tail collagen type I (1.3 mg/mL) and gelatin (7.5%) provided a relatively appropriate microenvironment for the growth of hepatocytes.

After the hepatocyte model was successfully established, the experiment verified the toxicity of emodin in bidirectional roles by using this model based on microfluidic chip. The results of 48 h emodin exposure experiment showed that the activity of the hepatocyte model decreased significantly with the increasing of emodin concentration, and the volume of hepatocyte in the cell culture chamber of chip gradually shrank and became smaller. This is consistent with the findings that large doses of emodin can induce apoptosis of L02 and HepG2 cells. [32,33]. However, compared with the cytotoxicity results obtained from these scholars on the basis of the conventional 96-well plate culture, the effective toxicity value of emodin was enhanced in the hepatocyte model based on microfluidic chip technique. Some literature results showed that emodin could inhibit significantly the activity of HepG2 cells when the concentration of that was larger than 15 μM [32,33], and other literature found that the significant inhibitory concentration of emodin on HepG2 cells was 50 μM [32], which were cultured as conventional technique (96 well plate culture) in these researches, while the initial significant inhibitory concentration of emodin was 100 μM in the challenge experiment on the hepatocyte model based on microfluidics chip. The reasons might be that the inhibition effects came from two aspects including emodin and metabolic waste of HepG2 cells, which co-existed in the culture fluid of conventionally static culture in previous researches, while microfluidic chip can dynamically and automatically implement perfusion for hepatocyte, and the metabolite of them can be eliminated out from the cell culture chamber to the waste fluid hole (the 7th hole) of chip, which can enable the hepatocyte model in microfluidic chip only to be affected by the toxicity of emodin. Therefore, the toxic dose of emodin was enhanced to 100 μM in hepatocyte model based on microfluidic chip. In addition, the cell staining results appeared with the same trend as the CCK-8 results. Without doubt, the above results need more verification in further experiments.

In the study, we evaluated the function and metabolism of the hepatocyte model after emodin exposure by using AST, LDH and BUN. Emodin exposure results showed that there were no significant differences in AST and LDH levels among different groups. As is known to all, the enzymology indexes of liver have changed although liver organ have not occurred organic changes in the early stage of hepatosis. AST, a common index of function evaluation of liver, will increase with the increasing of liver cell damage once liver function is damaged [34]. Although the AST did not increase significantly with the increasing of emodin concentration in our experiment, AST activity in the range of 50–200 μM showed an increasing trend compared with the blank group. In addition, it showed a decrease at 25 μM although that was not significant. The result of further analysis indicated that emodin has a certain protective effect on hepatocytes at low concentrations of 25 μM under microfluidic conditions. The above results are consistent with the results of emodin exposure experiments from many scholars based on conventional culture in 96-well plate for hepatocytes, which meant a small dose of emodin can afford a hepatoprotective role and cause the reduction of AST and ALT levels [31]. LDH, as a stable protein, will leak out from the hepatocyte once the hepatocyte membrane is damaged by toxicant [35], so LDH was also detected in this research to further determine the damage degree of hepatocyte after emodin exposure. However, LDH activity appeared to decrease with the increasing of emodin concentration from 0 to 200 μM in our experiment. Some researchers also encountered a similar situation, andthe problem also did not have an explanation [36]. On the other hand, some scholars once reported that LDH distributed respectively in the culture, apoptosis body and cell, and the calculation of the release rate of LDH could more accurately reflect the actual situation of the hepatocyte damage. But, the explicit explanation of the release rate was not tracked down until now [37]. In subsequent research, we will continue to focus on the change of LDH and conduct more in-depth research to explain this.

For the above results of relevant metabolic function indicators, some scholars once indicated that HepG2 cells, as a member of the hepatocyte line, did not express enough enzymes. Although most of these are phase I and II metabolic enzymes, not physiologically related [38,39,40,41], whether they also affect the activity and expression of other enzymes remains to be further studied. This is another guess about why there is no significant difference in the activities of LDH and AST in HepG2 cells 48 h after emodin exposure among emodin exposure groups and blank group.

When hepatocytes are damaged, BUN, as a secretion of healthy hepatocytes, would reduce [37]. The results of our research indicated that BUN significantly decreased with the increasing of emodin concentration, which is consistent with this theory.

We found that it is feasible and necessary to use the microfluidic chip technique to build a hepatocyte model in exploring emodin toxicity, which can improve the precision of cell culture through change culture fluid and simulate the environment of the human body by bionic method. In addition, the above feasibilities indicate that it will be possible to apply the constructed hepatocyte model in more aspects of evaluating the effects of raw materials of health food, which include not only the toxicity but also the nutritional benefits of them. For example, the constructed hepatocyte model also can be used to observe whether an ingredient of health food can prevent hepatic injury or improve hepatic function.

It also has been found that the above evaluation indicators need to be increased in the successive researches evaluating the effects of different substances of nutriology at a later stage, so as to explore more profound mechanisms that health foo may have on human health.

We hope this research can show more accurate and true evaluation effectiveness tp replace the results of conventional cell culture, and can provide references for more kinds of cells such as stomach cell, renal cell, myocyte, etc. which might be used to construct various types of models based on microfluidic chip technique, and can provide new schemes and basic information in more fields of nutriological evaluation in future.

## 5. Conclusions

The construction of a new type of hepatocyte model, which is based on the microfluidic chip technique, has been completed, and the application of that reveals a feasibile and bionic effect in evaluating emodin. Depending on the above results, the new hepatocyte model might be a potential replacement method for conventional cell experiments in evaluating the effects of the main ingredients of various raw materials of health food.

## Data Availability

The data supporting reported results can be obtained from the corresponding author.
